# Anti-CD30 chimeric antigen receptor T cell therapy for relapsed/refractory CD30^+^ lymphoma patients

**DOI:** 10.1038/s41408-020-0274-9

**Published:** 2020-01-23

**Authors:** D. Wang, C. Zeng, B. Xu, J.-H. Xu, J. Wang, L.-J. Jiang, Q.-X. Wang, C.-R. Li, N. Wang, L. Huang, Y.-C. Zhang, Y. Xiao, J.-F. Zhou

**Affiliations:** Department of Hematology, Tongji Hospital, Tongji Medical College, Huazhong University of Science and Technology, Wuhan, Hubei 430030 P.R. China

**Keywords:** Clinical trial design, Immunotherapy

Dear Editor,

Since human lymphoma cells have a specific antigen expression pattern according to cell types and stages of differentiation, immunotherapy has become a promising field in lymphoma treatment. CD30 is a type 1 transmembrane receptor that is consistently overexpressed on all stages of cells in classical Hodgkin lymphoma (HL) and anaplastic large-cell lymphoma (ALCL); meanwhile, on normal cells, CD30 expression is limited, indicating that CD30 is an ideal immunotherapeutic target for these lymphoma subtypes^1,2^. CD30 antibody–drug conjugate brentuximab vedotin (BV) has been applied either as a single agent or combining it with frontline regimens. Encouraging results with a high response rate and good safety profile were reported in newly diagnosed patients; on the other hand, in refractory/relapse (r/r) patients, although the overall reaction rate of BV was still impressive, the CR rate seemed unsatisfactory^3,4^. The same situation was faced when anti-PD-1 antibody was used in r/r HL patients^5,6^. Therefore, there is still a challenge to treat r/r patients with these targeted therapy drugs. Recently, a new immunotherapy technique, named chimeric antigen receptor (CAR) T cell therapy, was developed^7^. CAR-T therapy targeting CD19 has been reported with exciting results in r/r B cell acute lymphoblastic leukemia (B-ALL), and also demonstrated efficacy in r/r B cell non-Hodgkin lymphomas (B-NHL)^8,9^.

Based on the effectiveness of the CAR-T system and the specificity of CD30 expression, we designed a new third-generation anti-CD30 CAR, and conducted a pilot study to test the efficacy and safety of anti-CD30 CAR-T cell therapy on r/r CD30^+^ lymphoma patients. Study design, data collection, and analysis are described in the Supplementary Methods. This study design was approved by the institutional review board of Tongji Hospital, Tongji Medical College, and Huazhong University of Science and Technology, and registered with the Chinese Clinical Trial Registry (ChiCTR, number ChiCTR-OPN-16009069). All patients provided written informed consent before enrollment in accordance with the Declaration of Helsinki.

From 1 September 2016 to 1 November 2018, nine r/r patients, including six HL and three ALCL patients, were enrolled in this study (Supplementary Fig. S1). All the nine patients were confirmed with CD30^+^ lymphoma by immunohistochemistry. The previous regimens included multiple lines of chemotherapy, radiotherapy, or even autologous transplantation. However, the disease status before CD30 CAR-T infusion was two patients with partial remission (PR), two patients with stable disease (SD), and five patients with progressive disease (PD) (Table 1). Three of the PD patients had extremely high tumor burden with multiple extranodal involvements before infusion. The detailed clinical characteristics are shown in Supplementary Table S1. The patients were given the FC (fludarabin 25 mg/m^2^ and cyclophosphamide 20 mg/kg for 3 days) regimen for lymphodepletion 4 days before CAR-T cell infusion. According to previous reports, the dose of CD30 CAR-T cell could be up to 2 × 10^7^ per kg, which was much higher than that reported in CD19 CAR-T trials^10,11^. Besides, patients’ general conditions were also taken into consideration. As a result, a total dose of 0.7–3.2 × 10^7^ per kg of CAR-T cells was infused, with a median dose of 1.4 × 10^7^ cells per kg. No acute adverse events (AEs) were observed during CAR-T cell infusion. We observed manifestations of patients, and monitored the inflammatory cytokines continuously after CAR-T cell infusion.

**Table 1:** Clinical characteristics of patients.

Patient no.	Diagnosis stage at entry	Previous treatment	Disease status at entry	CAR^+^ T cells infused per kg	CRS grade	CRES	Best response	Current outcome
1	NS HL, II_B_	ABVD, BEACOPP, BV, DHAP, PD-1 antibody, RT, and ASCT	SD	1 × 10^7^	0	None	CR × 29 months	Relapsed, dead from infection
2	NS HL, II_A_	ABVD, PD-1 antibody, and RT	PD	1.15 × 10^7^	1	None	CR × 38+ months	Alive, NED
3	ALCL, II_B_	CHOP, CHOPE	PR	7 × 10^6^	0	None	CR × 28+ months	Alive, NED
4	NS HL, IV_EB_	ABVD, ICE, GDP, RT, and ASCT	PD	1.2 × 10^7^	5	None	NA	Dead from pleural hemorrhage
5	NS HL, IV_EB_	ABVD, GDP, DHAP, PD-1 antibody, and RT	PD	2 × 10^7^	2	None	CR × 10 weeks	Relapsed, alive
6	ALCL, IV_B_	CHOP, CHOPE, hyper-CVAD A + B, and RT	PD	3.2 × 10^7^	3	None	SD × 6 weeks	Dead from infection
7	ALCL, II_A_	CHOP, CHOPE, and DHAP	PR	2.45 × 10^7^	0	None	CR × 16 + months	Alive, NED
8	NS HL, III_EA_	ABVD, ICE, PD-1 antibody, and RT	SD	1.4 × 10^7^	1	None	CR × 11 months	Relapsed, alive
9	NS HL, II_B_	ABVD, R-DHAP, PD-1 antibody, and RT	PD	3.1 × 10^7^	1	None	CR × 13 months	Relapsed, alive

*ABVD* doxorubicin, bleomycin, vinblastine, dacarbazine, *ALCL* anaplastic large-cell lymphoma, *ASCT* autologous stem cell transplant, *BEACOPP* bleomycin, etoposide, doxorubicin, clophosphamide, vincristine, procarbazine, prednisone, *BV* brentuximab vedotin, *CHOP(E)* cyclophosphamide, doxorubicin, vincristine, prednisone (etoposide), *CR* complete remission, *CRES* CAR-T-related encephalopathy syndrome, *CRS* cytokine release syndrome, *DHAP* dexamethasone, high-dose cytarabine, cisplatin, *GDP* gemcitabine, dexamethasone, cisplatin, *hyper-CVAD A* cyclophosphamide, vincristine, doxorubicin, dexamethasone, *hyper-CVAD* ethotrexate, cytarabine, *ICE* ifosfamide, carboplatin, etoposide, *NED* no evidence of disease, *NS HL* nodular sclerosis Hodgkin lymphoma, *PR* partial remission, *PD* progressive disease, *RT* radiation therapy, *SD* stable disease.

Six (66.7%) patients experienced CRS, four were of low-grade (grade 1–2), and no CAR-T-related encephalopathy syndrome was observed in all the nine patients (Table 1). All AEs are evaluated in Supplementary Table S2. The three patients, who had higher tumor burden as mentioned, experienced more severe AEs. They experienced remittent high fever, hypotension, hypoxia, poor physical status, and elongated time to recovery from grade 3/4 cytopenia (Supplementary Table S3). They had obviously higher IL-6 and ferritin levels, from baseline to peak, throughout the treatment course (Fig. 1a, b). One (#4) of the three patients had progressive peritoneal and pleural effusion, in which a large amount of CD30 CAR-T cells was detected, implicating a continuous recruitment and expansion of CAR-T cells in tumor tissue. Unfortunately, he died at day 20, 3 h after an uncontrollable sudden pleural hemorrhage, which caused continuous hypotension and hypoxia. Given the fact that before hemorrhage, the pleural effusion was clear and CRS grading was grade 2 (Supplementary Table S4), we inferred that he died from hypovolemic shock and severe pulmonary atelectasis caused by the hemothorax. The source of hemorrhage might be an intratumoral vessel, since the patient had multiple pulmonary and pleural infiltration loci. On the other hand, the six patients who had lower tumor burden experienced only mild fever or nausea, and cytopenia was recovered within 2 weeks. Skin rash similar with inflammatory purpura was observed in two patients (Supplementary Table S2). No other AEs were recorded spanning the period of hospitalization. There was also no significant elevation of inflammatory cytokines for these six patients, with the maximum fold change from baseline less than five (Fig. 1a, b). Although the grade of AEs and CRS seemed parallel with tumor burden (Supplementary Fig. S2), most of the AEs were mild and controllable in general. The discrepancies of CRS inferred that tumor burden might be a risk factor of more severe CRS. In addition, the tumor infiltration of vital organs might result in lethal complications.

**Fig. 1:**
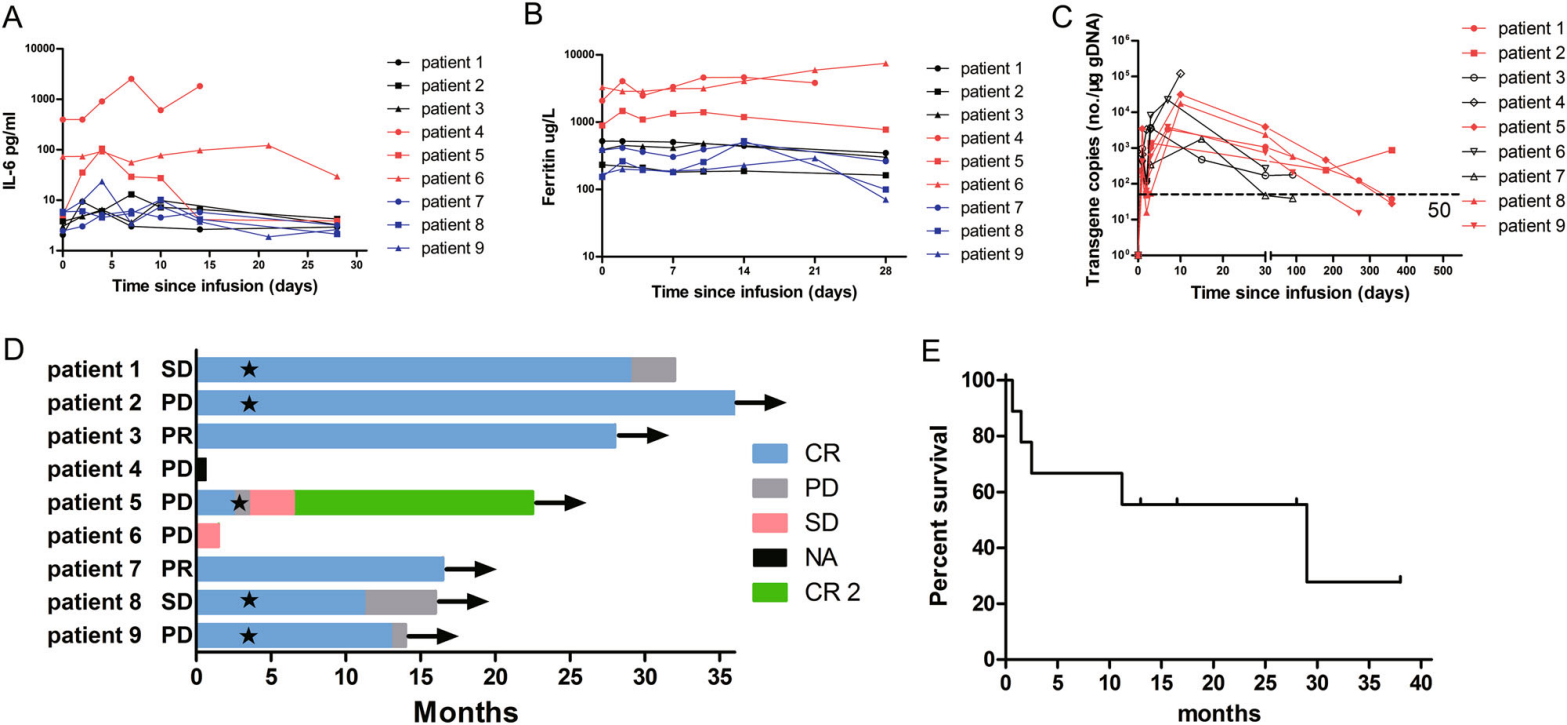
Patients’ characteristics and survival after CD30 CAR-T cell infusion. **a** The serum IL-6 level of each patient was assessed before and at serial time points after cell infusion; the red lines representing the three patients with higher tumor burden were much higher than the others. **b** The serum ferritin level of each patient was assessed before and at serial time points after cell infusion; the red lines representing the three patients with higher tumor burden were much higher than the others. **c** The copies of anti-CD30 CAR transgenes, the red lines representing the five patients who received anti-PD-1 antibody last longer; the horizontal line denotes the lower limit of quantitation (50 copies/μg). **d** Clinical responses for the nine patients. On the left of the *Y* axis is the disease status before infusion; arrows indicate alive; the star marker indicated the start time of anti-PD-1 antibody therapy. Patient #5 received autologous transplantation 4 months after PD, and achieved CR again (CR2). **e** Progression-free survival of all nine patients. NA not applicable, PD progression of disease, SD stable disease, PR partial response, CR complete response.

All patients were kept in the hospital after infusion, until there was neither evidence of infection nor leukocytopenia, and then returned monthly to have treatment response evaluated. The assessment of treatment response was carried out by CT/MRI/PET-CT scan at each visit. Excitingly, seven patients successfully achieved CR at the first visit. Relapse was observed in four patients (#5, #8, #9, and #1) after 10 weeks, 11, 13, and 28 months of CR, respectively (Fig. 1d). The median progression-free survival for all the nine patients was 13 months, with three long-term CRs over 2 years (Fig. 1d). The expansion of CAR-T cells was monitored by droplet digital polymerase chain reaction (ddPCR); most patients had persistent lentiviral copies for half a year (Fig. 1c). This result was hitherto one of the best responses to CD30 CAR therapy ever reported.

In comparison with the encouraging results achieved by CD19 CAR on B-ALL, there is little to report on anti-CD30 CAR-T cell therapy^10,11^. The results of existing phase I studies were unsatisfactory, with the best response being three complete remissions (CR) out of nine patients^10^. The reason for the poor response may be a lack of lymphodepletion, efficiency of CAR itself, or other unrevealed factors. In this study, we used FC regimen as lymphodepletion, which may, to some extent, exert slight tumor-reductive activities. However, we believed that the good response was due to our newly designed CD30 CAR, not the preceding chemotherapy, since all patients had refractory/relapse disease from previous cytotoxic chemotherapy (Supplementary Table S1). Our CD30 CAR contained two costimulatory domains from CD28 and 4-1BB (Supplementary Methods, Fig. S3). CAR-T cells by using CD28 as a costimulator have enhanced activation and faster proliferation, while 4-1BB endodomain results in a lower rate of T cell exhaustion, and can promote the persistence of CAR-T cells. The combination of the two costimulatory domains can both facilitate CAR-T cell proliferation, and elongate CAR-T cell existence in vivo^12,13^.

We also tested the possibility of combining anti-PD-1 antibody with anti-CD30 CAR-T cell therapy. Previous studies showed that anti-PD-1 antibody therapy had a high overall reaction rate, but a poor CR rate around 20% in phase II studies of r/r HL^14,15^. In our study, five of the HL patients had already received no less than three courses of anti-PD-1 antibody in previous treatment, but the response was two SD and three PD. After CD30 CAR infusion, anti-PD-1 antibody treatment was applied from day 90 or from the time when the patient was found to have progression of disease before day 90. Interestingly, progression of the relapsed patient (#5) was well controlled, and the other four patients remained in CR state for at least another 8 months after anti-PD-1 therapy started (Supplementary Table S5). The ddPCR also showed longer persistence of lentiviral copies in those five patients who received anti-PD-1 antibody therapy (Fig. 1c). These results indicated a synergetic effect of our CD30 CAR-T and the following anti-PD-1 antibody therapy.

Taken together, although the scale was limited, we showed the safety and efficacy of our CD30 CAR. We believe that our work will provide a new option for treatment of CD30^+^ lymphomas.

## Supplementary information

supplemental materials
