# Supplementary material for: Anti-CD30 chimeric antigen receptor T cell therapy for relapsed/refractory CD30+ lymphoma patients
Source: Blood Cancer J. 2020 Jan 23;10(1):8. doi: 10.1038/s41408-020-0274-9 (PMC6978321; doi:10.1038/s41408-020-0274-9)
Supplement: Supplementary file 1 — supplemental materials [file 41408_2020_274_MOESM1_ESM.docx]

Supplement to ***Di Wang, et al. Anti-CD30 Chimeric Antigen Receptor T Cell Therapy for Relapsed/Refractory CD30+ Lymphoma Patients***

This Supplementary Information has been provided by the authors to give readers additional information about their work.

**SUPPLEMENTARY INFORMATION**

**CONTENTS:**

[SUPPLEMENTARY METHODS 3](#_Toc16466417)

[Study design 3](#_Toc16466418)

[CAR construction 4](#_Toc16466419)

[Laboratory assessments 4](#_Toc16466420)

[Statistical analysis 4](#_Toc16466421)

[SUPPLEMENTARY TABLES 6](#_Toc16466422)

[Table S1. Supplemetary characteristics of patients at entry 6](#_Toc16466423)

[Table S2. Grading of adverse event recorded after CAR-T cell infusion 7](#_Toc16466486)

[Table S3. Details of events determining CRS grade, and time to recovery of cytopenia 8](#_Toc16466487)

[Table S4. Comparison of CRS grade and pleural effusions before and after pleural hemorrhage. 9](#_Toc16466504)

Table S5. Characteristics of the five Hodgkin’s lymphoma patients receiving anti-PD-1 therapy. **10**

[SUPPLEMENTARY FIGURS 11](#_Toc16466510)

Figure S1. Consort-like figure to show the enrollment of patients. 11

[Figure S2. Relationship between CRS grading and Ann Arbor staging. 12](#_Toc16466511)

[Figure S3. Schematic diagrams of CAR construct 13](#_Toc16466512)

[SUPPLEMENTARY REFERENCES 14](#_Toc16466513)

**SUPPLEMENTARY METHODS**

Study design

This study was approved by the institutional review board of Tongji Hospital, Tongji Medical College, Huazhong University of Science and Technology, and registered with the Chinese Clinical Trial Registry (ChiCTR, number ChiCTR-OPN-16009069, http://www.chictr.org.cn). All patients provided written informed consent before enrollment in accordance with the Declaration of Helsinki.

Eligible patients were ≥ 18 years old with good performance status (ECOG-PS ≤ 2) and essentially normal organ function; relapsed or refractory to their previous treatments, and had experienced at least two lines of systemic therapy, including autologous hematopoietic stem cell transplantation (auto-HSCT). The diagnoses were made according to the World Health Organization classification for tumors of the hematopoietic and lymphoid tissues. All patients were confirmed with CD30^+^ lymphoma by flow cytometry or immunohistochemistry. Key exclusion criteria included: active infection, another concomitant or prior malignancy, clinically evident neurological lesions, or pregnancy/breast-feeding.

The patients were given the FC (fludarabin 25mg/m^2^, cyclophosphamide 20mg/kg for 3 days) regimen as lymphodepletion(ref. [1](#_ENREF_1)) 4 days before CAR-T cell infusion. Anti-CD30 CAR T cells were infused on day zero. For Hodgkin’s lymphoma patients, treatment with pembrolizumab (100mg every 3 weeks) started at day 90 or at the time when the patient was found to have progression of disease before day 90. The anit-PD-1 therapy continued until progression or for eight cycles in patients without progression. Diagnostic imaging was performed for response assessment every month for half a year and every 3 months thereafter, including at least one PET-CT scan. All patients were followed up until they died, lost to follow-up, or withdrew consent. Cytokine release syndrome (CRS) was graded aaccording to the criteria from Lee et al.(ref. [2](#_ENREF_2)) CAR T-cell-related encephalopathy syndrome (CRES)(ref. [3](#_ENREF_3)) and other adverse events (AEs) were evaluated according to the National Cancer Institute Common Terminology Criteria for Adverse Events V5.0.

CAR construction

In this study we used the third-generation CAR which was composed of a single chain variable fragment (scFv), two costimulatory domains from CD28 and 4-1BB, and CD3ζ chain as activation domain (Supplementary Figure S3). The scFv was derived from a murine monoclonal antibody against human CD30.

Laboratory assessments

For patients who had bone marrow involvement, multiparameter flow cytometry (MFC) was used to quantitate minimal residual disease. In vivo expansion of CAR30 T cells were measured by droplet digital polymerase chain reaction (ddPCR), detailed procedure was as previously described (ref. [1](#_ENREF_1)). Cytokines were assessed according to the manufacturer’s instruction.

Statistical analysis

Progression-free survival (PFS) and overall survival (OS) were estimated as the time from infusion to first relapse or death (loss of follow up), respectively. Treatment response including complete remission (CR), partial remission (PR), stable disease (SD) and progression of disease (PD) were defined according to Younes et.al(ref. [4](#_ENREF_4)). The analysis of categorical variables was performed using Fisher’s exact test for 2 × 2 tables. All calculations were performed using SPSS software version 16.0 (SPSS, Chicago, IL). *P* values less than 0.05 (two-tailed) were considered to be statistically significant.

**SUPPLEMENTARY TABLES**

Table S1. Supplemetary characteristics of patients at entry

| Patient No. | Age (years) | Sex | Extranodal Sites | IPI score | Previous treatment response | Response to last cytotoxic therapy |
| --- | --- | --- | --- | --- | --- | --- |
| 1 | 29 | M | No | 3 | 5th relapse | PR |
| 2 | 23 | M | No | 2 | Primary refractory | PD |
| 3 | 46 | M | No | 1 | First relapse | PR |
| 4 | 31 | M | lung, pleura, liver, abdominal mass | 4 | Second relapse | PD |
| 5 | 22 | F | lung, pleura, subcutaneous mass | 4 | Third relapse | SD |
| 6 | 20 | F | Bone marrow | 3 | Primary refractory | PD |
| 7 | 26 | M | No | 2 | First relapse | PR |
| 8 | 36 | F | breast, pericardium | 3 | Primary refractory | PR |
| 9 | 20 | F | No | 1 | Second relapse | SD |

| Adverse event | Number of patiens | | | | |
| --- | --- | --- | --- | --- | --- |
|  | Grade 1 | Grade 2 | Grade 3 | Grade 4 | Grade 5 |
| Fever | 3 | 1 | 2 | 0 | 0 |
| Headache | 4 | 1 | 0 | 0 | 0 |
| Hypoxia | 0 | 0 | 1 | 0 | 1 |
| Pulmonary edema | 0 | 0 | 1 | 0 | 0 |
| Pleural hemorrhage | 0 | 0 | 0 | 0 | 1 |
| Hypotension | 1 | 1 | 0 | 0 | 1 |
| Tachycardia | 1 | 1 | 0 | 0 | 0 |
| Nausea | 5 | 1 | 1 | 0 | 0 |
| Vomiting | 1 | 1 | 0 | 0 | 0 |
| Oral mucocitis | 2 | 1 | 0 | 0 | 0 |
| Purpura | 0 | 2 | 0 | 0 | 0 |
| Leukopenia | 0 | 1 | 3 | 5 | 0 |
| Neutropenia | 0 | 2 | 3 | 4 | 0 |
| Anemia | 4 | 0 | 2 | 2 | 0 |
| Thrombocytopenia | 2 | 2 | 1 | 1 | 0 |
| Prolonged APTT | 1 | 0 | 0 | 0 | 0 |
| Hyperuricemia | 1 | 0 | 0 | 0 | 0 |
| Transaminitis | 1 | 0 | 0 | 0 | 0 |
| Hypoalbuminemia | 3 | 1 | 1 | 0 | 0 |

Table S2. Grading of adverse event recorded after CAR-T cell infusion

Table S3. Details of events determining CRS grade, and time to recovery of cytopenia

| Patient No. | Temperature peak, ℃ | Hypotension | Hypoxia | Time to recovery of cytopenia, days |
| --- | --- | --- | --- | --- |
| 1 | 37.0 | No | No | 22 |
| 2 | 38.5 | No | No | 12 |
| 3 | 37.0 | No | No | 10 |
| 4 | 39.1 | Requiring multiple vasopressors | Requiring mechanical ventilation | No recovery at death (day20) |
| 5 | 39.6 | Not requiring vasopressors | Requiring low flow nasal cannula | 15 |
| 6 | 40.1 | Requiring one vasopressor | Requiring low flow nasal cannula | 27 |
| 7 | 36.8 | No | No | 12 |
| 8 | 38.6 | No | No | 8 |
| 9 | 39.3 | No | No | 11 |

Table S4. Comparison of CRS grade and pleural effusions of patient #4 before and after pleural hemorrhage.

| Patient Characteristics | D17 | D20 |
| --- | --- | --- |
| Temperature peak, ℃ | 37.6 | 37.9 |
| Hypotension | No | Requiring multiple vasopressors |
| Hypoxia | Requiring low flow nasal cannula | Requiring mechanical ventilation |
| CRS grade | 2 | 4 |
| Hemoglobin | 80g/L | 49g/L |
| Color of pleural effusion | yellow | Color of venous blood |
| Rivalta test of pleural effusion | positive | positive |
| Erythrocyte count in pleural effusion | 8.48×10^9^/L | 1057×10^9^/L |
| Karyocyte count in pleural effusion | 190×10^6^/L | 220×10^6^/L |
| Percentage of neutrophil in pleural effusion | 4 | 33 |
| Percentage of lymphocyte in pleural effusion | 88 | 62 |

Table S5. Characteristics of the five Hodgkin’s lymphoma patients receiving anti-PD-1 therapy.

| Patient No. | Best response to prior anti-PD-1 therapy before infusion | Time of start | Disease status at strat | Adverse events | Best response | Time to progression |
| --- | --- | --- | --- | --- | --- | --- |
| 1 | SD | Day 90 | CR | NO | CR | 26 months |
| 2 | PD | Day 90 | CR | NO | CR | 35+ months  (No progression) |
| 5 | PD | Day 70 | PD | NO | SD | NA |
| 8 | SD | Day 90 | CR | NO | CR | 8 months |
| 9 | PD | Day 90 | CR | NO | CR | 10 months |

**SUPPLEMENTARY FIGURS**

**Figure S1. Consort-like figure to show the enrollment of patients.**


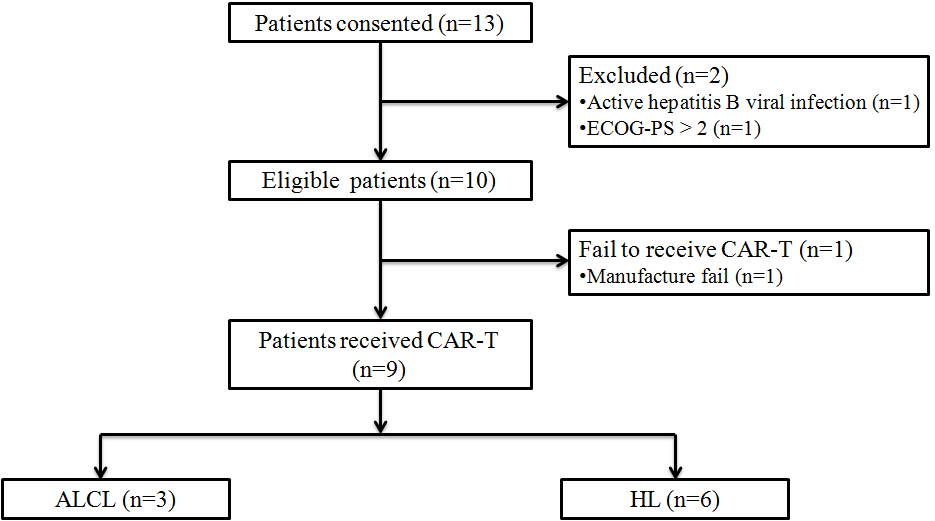


**Figure S1. Consort-like figure to show the enrollment of patients.** A total of twelve patients were consented. Two patients were excluded during screening because they didn’t meet the criteria. One eligible patient finally failed to receive CAR T therapy because of unsuccessful manufacture of CAR T cell for three times. The remaining nine patients were three ALCL and six HL patients. Abbreviations: ECOG-PS: performance status by Eastern Cooperative Oncology Group; ALCL: anaplastic large cell lymphoma; HL: Hodgkin’s lymphoma.

Figure S2. Relationship between CRS grading and Ann Arbor staging.


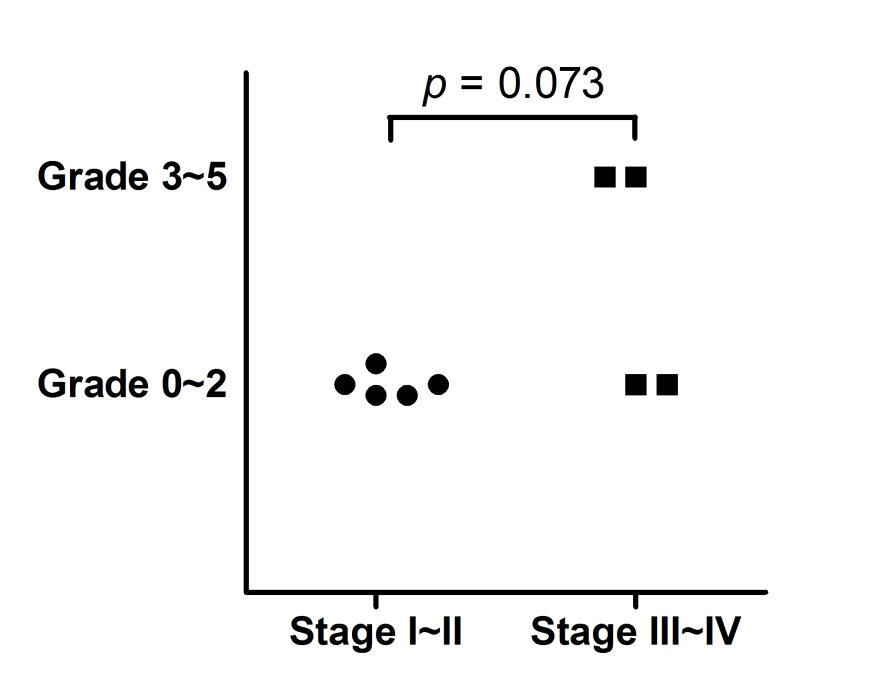


**Figure S2. Relationship between CRS grading and Ann Arbor staging.** Patients with Ann Arbor staging III~IV seemed to have higher risk of more severe CRS than patients with stage I~II, however the difference was not statistically significant (p = 0.073).

Figure S3. Schematic diagrams of CAR construct


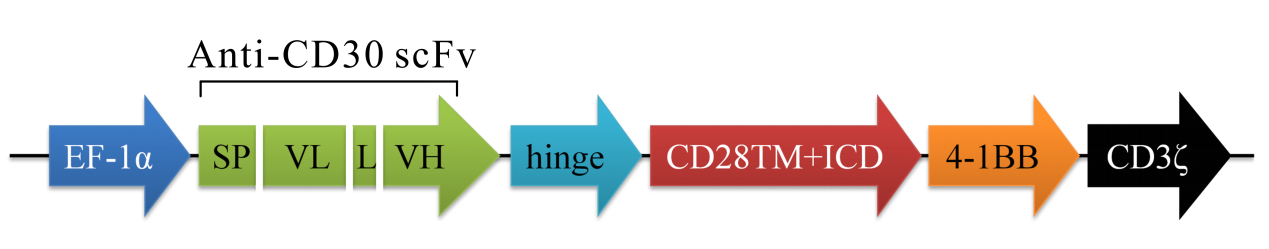


**Figure S3. Schematic diagrams of CAR construct.** The third-generation CAR was composed of a single chain variable fragment (scFv), two costimulatory domains from CD28 and 4-1BB, and CD3ζ chain as activation domain. The scFv was derived from a murine monoclonal antibody against human CD30. Abbreviations: SP, signal peptide; VL, variable L chain; L, linker; VH, variable H chain.

**SUPPLEMENTARY REFERENCES**

1. Wang N*, et al.*, Efficacy and safety of CAR19/22 T-cell cocktail therapy in patients with refractory/relapsed B-cell malignancies*.* *Blood*, 2020. 135(1): p. 17-27.

2. Lee DW*, et al.*, ASTCT Consensus Grading for Cytokine Release Syndrome and Neurologic Toxicity Associated with Immune Effector Cells*.* *Biol Blood Marrow Transplant*, 2019. 25(4): p. 625-638.

3. Neelapu SS*, et al.*, Chimeric antigen receptor T-cell therapy - assessment and management of toxicities*.* *Nat Rev Clin Oncol*, 2018. 15(1): p. 47-62.

4. Younes A*, et al.*, International Working Group consensus response evaluation criteria in lymphoma (RECIL 2017)*.* *Ann Oncol*, 2017. 28(7): p. 1436-1447.
